# Adjuvant immunotherapy for muscle-invasive urothelial carcinoma: Considerations for targeting patients

**DOI:** 10.1177/23523735261424950

**Published:** 2026-03-16

**Authors:** Sarafina Urenna Otis, Aruni Ghose, Giuseppe Luigi Banna, Akash Maniam

**Affiliations:** 1 School of Medicine and Biomedical Sciences, 6396 University of Oxford, John Radcliffe Hospital, Oxford, England; 2 Barts Cancer Centre, 112003 St Bartholomew's Hospital, Barts Health NHS Trust, London, England; 3 Cancer Research UK City of London Centre, Queen Mary University of London, 105710 Barts Cancer Institute, London, England; 4 Health Systems and Treatment Optimisation Network, 242021 European Cancer Organisation, Brussels, Belgium; 5 6698 Portsmouth Hospitals University NHS Trust, Portsmouth, England; 6 Faculty of Science and Health, 14303 School of Pharmacy and Biomedical Sciences, University of Portsmouth, Portsmouth, England

**Keywords:** MIUC, urothelial cancer, adjuvant, immunotherapy, biomarker, anti-PD-L1, ctDNA, patient targeting

## Abstract

While neoadjuvant chemotherapy and radical surgery represent mainstay treatments for muscle-invasive urothelial carcinoma (MIUC), recurrence with lethal metastasis remains high and highlights the need for adjuvant therapies in MIUC, like immunotherapy, already in use for metastatic UC with favourable results. This review provides an overview of the key clinical trials investigating adjuvant immune checkpoint inhibitors (ICIs) for MIUC and their clinical implications; in particular, examining factors that may be relevant in guiding adjuvant therapy to patients, such as ICI therapy choice, tumour subtype and the clinical utility of biomarkers, specifically PD-L1 status and circulating tumour DNA (ctDNA). Of the three key trials CheckMate-274, IMvigor010 and AMBASSADOR, anti-PD1 inhibitors nivolumab and pembrolizumab have shown statistically significant improvements in disease-free survival (DFS) compared to controls, highlighting their promising use as seen with nivolumab's approval into clinical practice. Results are conflicting on the association of PD-L1 tumour expression with treatment outcome, yet ctDNA has emerged as a key biomarker from IMvigor010, showing not only prognostic value but also association between its clearance and atezolizumab treatment benefit derived. Notably, it remains uncertain across the trials whether adjuvant treatment efficacy differs by tumour origin (upper tract or lower tract disease), and larger subgroup numbers are needed for future trials in order to adequately assess statistical significance of speculative associations. Altogether, study findings support increasing clinical incorporation of adjuvant nivolumab and pembrolizumab into the MIUC setting, and emphasise ctDNA's utility as a biomarker for MIUC, demonstrating roles in both prognostication and prediction of treatment benefit.

## Introduction

Urothelial carcinoma (UC) represents the 9th most common cancer worldwide, and is categorised into upper tract urothelial carcinoma (UTUC), i.e., urothelial cancer of the renal pelvis and ureter, or cancer of the lower urinary tract (bladder and urethra), out of which bladder cancer (BC) constitutes the most common subtype, representing around 90% of all UCs.^1–3^ The stage of invasiveness is another key parameter in UC, with muscle-invasive urothelial carcinoma (MIUC) representing a clinically significant stage associated with higher relapse and mortality post-treatment.^2,4,5^ MIUC accounts for a notable total proportion of urothelial cancers, representing around 25–30% of UCs at presentation (T2 and higher), with a further 40% of the remaining high-risk, non-muscle invasive cancers (Ta/T1) going on to progress to muscle invasion.^5,6^ In addition, despite the lower incidence of UTUC (∼10%) compared to BC, MIUC is over-represented in patients with UTUC, with around 60% of patients that present with UTUCs having muscle-invasive disease at the point of diagnosis compared to 15–20% of patients that are diagnosed with BC.^2,7,8^ Thus, MIUC presents an area of significant clinical burden within the field of urothelial cancers.

Despite current mainstay treatments, treatment outcomes for patients with MIUC remain suboptimal. Radical nephroureterectomy (RNU) with bladder cuff excision forms the gold standard for the treatment of patients with high-risk, non-metastatic UTUC disease.^3^ For lower-tract BC, alongside radical cystectomy, neoadjuvant cisplatin-based chemotherapy (NAC) is part of the standard care received for patients diagnosed with non-metastatic, locally advanced UC, following studies in the 2000s that demonstrated clinically meaningful improvements in survival with the addition of NAC compared to surgery alone.^2,3,9,10^ Yet up to 50% of relevant patients are cisplatin-ineligible, precluding these individuals from treatment with neoadjuvant chemotherapy, and even among the patients receiving standard care, MIUC is still associated with high recurrence risk and increased mortality post-relapse, with trial findings approximating 30% of the patients to experience metastatic recurrence within 2 years of diagnosis.^2,5,11^ Therefore, a need exists for additional systemic therapies to help improve clinical outcomes for MIUC, especially in the adjuvant setting where eradication of possible micrometastases may help in the prevention of distant metastases that have been shown to associate with lethality of recurrence post-surgery in MIUC.^12^

While adjuvant chemotherapy presents one form of additional systemic therapy for MIUC that already sees occasional use in this setting, challenges remain due to a lack of consensus on the routine use of adjuvant cisplatin-based chemotherapy for MIUC, data inconsistency for evaluation of outcomes in patients with residual disease following standard care, as well as the persisting problem of cisplatin ineligibility in patient cohorts.^7,13–15^ Instead, there has been a growing focus on the use of immunotherapy, which has already seen increasing incorporation into routine care for metastatic UC, and the question of whether benefits with adjuvant immune checkpoint inhibitor (ICI) therapy might also translate to the MIUC setting.^16–18^ A number of key trials have thus far explored the use of adjuvant immunotherapy following surgical resection with or without NAC in patients with MIUC, notably CheckMate-274, IMvigor010 and Alliance A031501 AMBASSADOR, which investigated the use and clinical efficacy of nivolumab, atezolizumab and pembrolizumab, respectively, and provided results on the key clinical endpoints of disease-free survival (DFS) and overall survival (OS) where available.^19–23^ As some of the first trials investigating adjuvant immunotherapy use in this setting, their respective trial outcomes are important to examine considering the potential for clinical translation to MIUC, as already seen with regulatory approvals for nivolumab and, less widely, pembrolizumab.^24–28^

More so, focus on the improvement of treatment outcomes for patients with MIUC has not only led to the exploration of new adjuvant immunotherapies but also questions on how adjuvant therapy may be appropriately targeted to patients, aiming to increase clinical efficacy while minimising the experience of toxic side-effects by patients that are less likely to benefit of these therapies. This is of particular importance to consider given the current uncertainty around which populations truly benefit from ICI treatment use, which is also evident in the metastatic UC setting.^29^ The use of subgroups in the aforementioned trials provides opportunity for examining relevant factors that may impact adjuvant treatment outcomes for MIUC populations.^19–23^ Tumour subtype (UTUC or lower tract disease) is one such factor of interest, for which subgroup analyses of patients may highlight any differential efficacy of adjuvant immunotherapy use, important for future clinical consideration of guiding treatment choice for patients. The use of tumour biomarkers in UC presents another area of interest, not only for prognostic applications, but also for potential use in the prediction of treatment effects, which is also beneficial in informing appropriate treatment guidance. Histological analysis of PD-L1 tumour expression has been a key metric used for several cancers in tumour characterisation as well as for prognosis and/or prediction of treatment effect, particularly in the context of ICI treatment that targets immune checkpoint regulators, like PD-L1.^30^ While PD-L1 has been shown to provide prognostic value in metastatic urothelial carcinoma, its utility as a biomarker in MIUC had until recently been under-examined, with results from the key adjuvant immunotherapy trials providing a new opportunity for its assessment as a predictive biomarker in MIUC.^19–23^^,31^ Moreover, circulating tumour DNA (ctDNA), most commonly derived from plasma, has emerged as a promising biomarker among numerous cancers, notably lung cancer and colorectal cancer, with interest also developing within the MIUC setting to examine whether this non-invasive, accessible strategy may be applicable for predicting adjuvant treatment response and/or helping to monitor and identify recurrence in these high-risk patients.^21,22,32–34^

Altogether, this review aims to explore the use of adjuvant immunotherapy for high-risk, muscle-invasive urothelial carcinoma, highlighting the key trials to date, alongside the populations shown to most benefit from such perioperative ICI treatment. It will offer a discussion on key areas that may influence how adjuvant therapy should be guided, including tumour subtype (UTUC versus BC) and biomarker application, notably the expanding use of ctDNA, alongside their impact on clinical efficacy and therapy outcomes.

## Key trial results for adjuvant immunotherapy use in MIUC

### CheckMate-274

CheckMate-274 was the first phase 3, randomised controlled trial in the MIUC setting to investigate and demonstrate the efficacy of adjuvant ICI use, specifically the use of nivolumab as an adjuvant therapy in patients with MIUC at high risk of metastatic recurrence following standard treatment of radical surgery with or without NAC.^19,20^ Investigations into the use of adjuvant nivolumab for MIUC ensued following the increasing application of nivolumab in the metastatic UC setting, available to patients subsequent to initial treatment with platinum-based chemotherapy.^35,36^ A total of 709 patients with MIUC who had undergone previous radical surgery were included in CheckMate-274, which was eligible to MIUC patients at high risk of recurrence, defined as having a pathological tumour stage of ypT3 to ypT4 or lymph node positivity (ypN+) in those who had received prior NAC, or as pT3-pT4a or pN + in patients without prior NAC who were not eligible for or declined adjuvant cisplatin chemotherapy.^19^ The trial compared the use of adjuvant nivolumab versus placebo, given every 2 weeks up to 1 year, between patient groups, examining the impact on the primary endpoints of DFS in the intention-to-treat (ITT) population and patients with tumour PD-L1 >=1%, as well as the secondary endpoints of OS and non-urothelial tract recurrence-free survival (NUTRFS). Trial results from pre-specified interim analyses showed promising results of adjuvant nivolumab use; data analysis of the intention-to-treat (ITT) population revealed a median DFS of 20.8 months (95% CI, 16.5–27.6) in patients who received nivolumab compared with 10.8 months (95% CI, 8.3,13.9) in the control group, demonstrating a 30% reduced risk of recurrence of disease or death with adjuvant nivolumab use (HR 0.70; 95% CI, 0.57–0.86, p = 0.0008).^19^ Longer DFS was also shown to be associated with ICI treatment among patients with a tumour PD-L1 expression level of 1% or more: at that timepoint of interim analysis, median DFS had not been reached in PD-L1 >=1% patients receiving nivolumab (95% CI; 21.2, not estimable) whereas patients on placebo saw a shorter DFS of 8.4 months (95% CI, 5.6–21.2) (HR 0.55; 95% CI, 0.39–0.77, p = 0.0005).^19^ Results of these initial interim analyses are particularly important to highlight given the seminal impact these findings had in accelerating regulatory approval for the use of adjuvant nivolumab for MIUC patients following radical surgery, a first of such ICI therapies within the setting. While the Food and Drug Administration (FDA), in being the first to process approval of adjuvant nivolumab in 2021, offered eligibility to all patients with high recurrence risk UC undergoing radical resection, later approvals under the European Commission have restricted the use to MIUC patient cohorts with a PD-L1 tumour status of 1% or more, taking into consideration subgroup data analysis from the CheckMate-274 trial.^24,25^ The National Institute for Health and Care Excellence (NICE) is another organisational body that has recommended adjuvant nivolumab for the treatment of MIUC that is at high risk of recurrence after radical resection, limited to adults whose tumours express PD-L1 at a level of 1% or more where adjuvant treatment with platinum-based chemotherapy is unsuitable.^26^

Importantly, the latest CheckMate-274 update, reporting 3-year extended follow-up results together with the first report of OS data in the trial thus far, continues to show improvements in clinical outcomes with adjuvant nivolumab use.^20^ Consistent DFS benefit is seen with nivolumab over placebo use as in previous interim analyses, demonstrating a 29% reduction in risk of recurrence or death (HR 0.71; 95% CI, 0.58–0.86).^20^ Current OS data shows a similar trend, with greater OS benefit seen for nivolumab-treated patients than those with placebo (HR 0.76; 95% CI, 0.61–0.96), especially among the patients with tumour PD-L1 >=1%, who see a 44% reduction in risk of death compared to control groups (HR 0.56; 95% CI, 0.36–0.86) (Table 1).^20^ Altogether, these DFS and OS findings continue to support adjuvant nivolumab as a standard of care for high-risk MIUC after radical resection, in addition to validating previous observations that DFS highly correlates with OS in MIUC. However, unlike PD-L1 status, which is shown for patient groups with PD-L1 >=1% to be associated with greater clinical benefit, subgroup analysis based on tumour origin in CheckMate-274 is only speculative. Based on subgroup data, no clear benefit in OS for UTUC cancers (N = 74) was seen compared to control groups (N = 75) (renal pelvis: HR 0.67; 95% CI, 0.31–1.46 and ureter: HR 3.93; 95% CI, 1.20–12.88) whereas possible benefit of adjuvant nivolumab over placebo may be suggested by subgroup data for patients with lower tract bladder cancer (N = 279) (HR 0.7; 95% CI, 0.55–0.90).^20^ However, notable limitations are present, including non-prespecification of the subgroups for analysis and indeterminate patient numbers that are needed for adequate statistical powering, and, overall, lack of statistical significance of the findings cautions against over-interpretation of the findings on differential efficacy of adjuvant nivolumab by tumour subtype for MIUC patients.

**Table 1.** Summary of the study demographics, clinical endpoints and outcomes from the key phase 3, RCTs, CheckMate-274, IMvigor010 and AMBASSADOR, investigating adjuvant ICI use for MIUC.

		CheckMate-274 *(Galsky et al., 2025)*^20^		IMvigor010 *(Powles et al., 2021 & 2024)*^21,22^		AMBASSADOR *(Apolo et al., 2025)*^23^	
Treatment/control arm		Nivolumab	Placebo	Atezolizumab	Observation	Pembrolizumab	Observation
Inclusion criteria		NAC-ve: pT3-4a or pN+				NAC-ve: pT3-4a or pN + or PSM	
		NAC+ve: ypT2-4a or ypN+				NAC+ve: ypT2-4a or ypN+ or PSM	
Number of patients		353	356	406	403	354	348
Primary tumour site, n (%)	Upper tract disease	74 (21)	75 (21)	29 (7)	25 (6)	81 (23)	72 (21)
	Lower tract disease	279 (79)	281 (79)	377 (93)	378 (94)	273 (77)	276 (79)
Positive PD-L1 status, n (%)		140 (40)	142 (40)	196 (48)	196 (49)	202 (57)	201 (58)
Primary endpoint		DFS		DFS		DFS, OS	
Secondary endpoint		OS, DSS, NUTRFS		OS, DSS, DMFS, NUTRFS		DFS, OS stratified by PD-L1 status	
Median DFS, months (95% CI)	ITT	22.0 (18.8–36.9)	10.9 (8.3–15.2)	19.4 (15.9–24.8)	16.6 (11.2–24.8)	29.6 (20.0–40.7)	14.2 (11.0–20.2)
HRs for DFS	ITT	0.71 (0.58–0.86)		0.89 (0.74–1.08)		0.73 (0.59–0.90)	
	Positive PD-L1 status	0.52 (0.37–0.72)		1.01 (0.76–1.35)		0.81 (0.61–1.08)	
	Upper tract disease	Renal pelvis: 1.22 (0.69–2.15); Ureter: 1.41 (0.65–3.06)		1.25 (0.57–2.74)		Renal pelvis: 1.96 (0.92–4.17); Ureter: 0.77 (0.29–2.02)	
	Lower tract disease	0.63 (0.51–0.78)		0.91 (0.74–1.10)		0.66 (0.53–0.83)	
Median OS, months (95% CI)	ITT	69.5 (58.1-NE)	50.1 (38.2-NE)	61.4 (46.5-NE)	59.0 (46.6-NE)	50.9 (43.8-NR)	55.8 (53.3-NR)
HRs for OS	ITT	0.76 (0.61–0.96)		0.91 (0.73–1.13)		0.98 (0.76–1.26)	
	Positive PD-L1 status	0.56 (0.36–0.86)		1.04 (0.74–1.46)		n/a	
	Upper tract disease	Renal pelvis: 0.67 (0.31–1.46); Ureter: 3.93 (1.20–12.88)		n/a		n/a	
	Lower tract disease	0.71 (0.55–0.90)		n/a		n/a	

Where positive PD-L1 status is defined as TPS PD-L1 >=1%, IC2/3 and positive CPS, respectively, for CheckMate-274, IMvigor010 and AMBASSADOR.

*RCT* randomised controlled trial, *MIUC* muscle-invasive urothelial carcinoma, *TPS* tumour proportion score, *PD-L1* programmed death-ligand 1, *IC* immune cell score, *NAC* neoadjuvant chemotherapy, *PSM* positive surgical margins, *CI* confidence interval, *DFS* disease-free survival, *OS* overall survival, *DSS* disease-specific survival, *DMFS* distant metastasis-free survival, *NUTRFS* non-urinary tract recurrence-free survival, *ITT* intention-to-treat (population), *NE* not estimable, *NR* not reached

### Alliance A031501 AMBASSADOR

Alliance A031501, the AMBASSADOR study, was another key trial to test adjuvant immunotherapy use among MIUC patients, investigating the use of pembrolizumab as an adjuvant therapy post radical surgery.^23^ Pembrolizumab had already been approved as a monotherapy and in combination with antibody-drug-conjugates enfortumab vedotin for the treatment of patients with metastatic urothelial carcinoma, providing clinical rationale for exploring whether its use could extend to earlier UC settings.^33,34^ In this phase 3, randomised trial, a total of 702 patients with high-risk MIUC after radical surgery (>=ypT2 or ypN + or microscopic + ve surgical margins in patients with prior NAC, >=pT3 or pN + or microscopic + ve surgical margins in patients without NAC) were enrolled between 2017 and 2021, and were randomly assigned to receive pembrolizumab or observations every 3 weeks for up to 18 cycles The main clinical endpoints to be assessed at interim/final analyses were the primary endpoints of DFS and OS, stratified by PD-L1 status as secondary trial endpoints (Table 1).^23^ Notably, the trial was closed early due to FDA approval of adjuvant nivolumab for MIUC, meaning that accrual was limited to 96% of the planned 734 patients to be enrolled and that continued observation in the control arm became inappropriate due to availability of approved adjuvant ICI therapy.^23,24^ Despite early closure, at the latest trial update, with a median follow-up duration of 44.8 months, findings, in line with adjuvant ICI benefit demonstrated by CheckMate-274, show that adjuvant pembrolizumab compared with observations alone was associated with significant DFS prolongation in the ITT population, demonstrating a doubling of median DFS survival with pembrolizumab (29.6 months; 95% CI, 20.0–40.7) over observation (14.2 months; 95% CI, 11.0–20.2) (HR 0.73; 95% CI, 0.59–0.90; p = 0.0027). This DFS benefit was observed regardless of PD-L1 status: PD-L1 + ve tumour status was not shown to be predictive of DFS benefit compared to the observation group (HR 0.81; 95% CI, 0.61–1.08).^23^ In addition, despite subgroup data, based on primary tumour site, suggesting a trend of DFS benefit with use of adjuvant pembrolizumab in MIUC of lower tract origin over control groups, (HR 0.66; 95% CI, 0.53–0.83), not similarly seen for subgroup UTUC data (see Table 1), lack of statistical significance of the data invalidates any definitive findings.^23^ Of further importance to note regarding the AMBASSADOR trial is that OS, being a key trial endpoint, to date remains immature, preventing final analyses of OS data as crossing of efficacy boundaries required for such is awaited (Table 1).^23^ Nonetheless, recent updates to the US's National Comprehensive Cancer Network (NCNN) guidelines feature increasing adoption of adjuvant pembrolizumab, specifically for patients with high-risk muscle-invasive bladder cancer (MIBC) (p3, pT4 or PN + disease) who have not undergone prior NAC.^27,28^ Thus far, the approval and recommendation of adjuvant pembrolizumab for MIBC is limited to the US, in comparison to more widespread international adoption of adjuvant nivolumab for MIUC, given notable limitations of the AMBASSADOR trial and its findings.^24–28^ These include OS immaturity and confounding based on off-trial ICI use in the observation arm as well as uncertainty around the differential efficacy of adjuvant pembrolizumab and restrictions in its use based on tumour subtype.^23^ Therefore, while adjuvant pembrolizumab use demonstrates clear DFS benefit, as seen with its previous interim analyses, whether it also confers a survival benefit in patients with MIUC in the adjuvant setting and may lead to wider regulatory approvals remains to be seen.

### IMvigor010

Soon after early analyses from CheckMate-274, results from IMvigor010 investigating the adjuvant use of anti-PDL1 inhibitor atezolizumab among MIUC patients were also published. Unlike the two other key trials, however, the phase 3 IMvigor010 trial did not meet its primary endpoint of improved DFS in the atezolizumab group over observation (Table 1).^21^ 809 patients with high-risk MIUC, defined as having ypT2-ypT4a or ypN + tumours following NAC or pT3-pT4a or pN + tumours in patients without prior NAC, were enrolled in the trial and received either atezolizumab or observations every 3 weeks for 16 cycles or up to 1 year. Data from the first interim analysis shows that there was no statistically significant difference in DFS between the treatment and control group (stratified HR 0·89 [95% CI 0·74–1·08], log-rank p = 0·24), failing to show any DFS benefit with adjuvant atezolizumab use over observations. Data for OS, a secondary endpoint of the trial, was still immature and to be awaited at further analyses.^21^ The latest 2024 update at 46.8 months median follow-up provides not only ad hoc analysis of the updated OS data in the ITT population but also data on subgroups defined by baseline ctDNA status, a key biomarker for which assessment was a prespecified exploratory endpoint in the study not included in the first interim report (Table 2).^22^ Updated trial data follows earlier interim results, finding that atezolizumab did not improve OS versus observation in the ITT population (HR 0.91; 95% CI, 0.73–1.13),.^22^ However, the exploratory analysis of ctDNA status included in the trial update demonstrates ctDNA positivity in MIUC as a key parameter shown to predict benefit with atezolizumab, in addition to having prognostic use, exemplified by the link between ctDNA positivity and poorer prognosis in the observation arms (HR 6.3; 95% CI, 4.3–9.3). Notably, ctDNA + ve patients receiving treatment with atezolizumab demonstrated an OS benefit favouring atezolizumab over observation (HR 0,59, 95% CI, 0.42–0.83), with longer OS outcomes seen for patients that achieved greater reductions in ctDNA levels under treatment.^22^ Additionally, longer OS was seen in the treatment groups over observation for patients with higher tumour PD-L1 expression, categorised as IC2/3 (HR 0.51; 95% CI, 0.30–0.85), contrasting previous updates that showed that higher PD-L1 expression was only associated with positive prognostication but no greater treatment benefit.^22^ However, while these findings appear promising, interpretations of an exploratory endpoint from negative trial results must be exercised with caution and more so justify further investigations into the trial endpoint. Therefore, although atezolizumab was initially shown to produce no clinical benefit over control in patients with high-risk MIUC, under higher PD-L1 status and/or ctDNA positivity subgroups, use of the adjuvant ICI has been shown to demonstrate statistically significant improvements in OS data, identifying a clinically meaningful application for adjuvant atezolizumab in the setting of MIUC.

**Table 2.** Summary of updated OS data by baseline ctDNA status provided from exploratory ad-hoc analysis of IMvigor010, a key phase 3 RCT investigating adjuvant atezolizumab use versus observation in patients with MIUC.

		IMvigor010 *(Powles et al., 2021 & 2024)* ^21,22^			
Subgroup (baseline)		ctDNA + ve		ctDNA -ve	
Treatment/control arm		Atezolizumab	Observation	Atezolizumab	Observation
Inclusion criteria		NAC-ve: pT3-4a or pN+			
		NAC+ve: ypT2-4a or ypN+			
BEP, n (%)		214 (37)		367 (63)	
Exploratory endpoints		OS by ctDNA, PD-L1 status			
Median OS, months (95% CI)	BEP	29.8 (20.7–40.2)	14.1 (10.5–19.7)	n/a	NR (NE)
	PD-L1 IC0/1	18.7 (12.4-34.2)	16.3 (10.4-20.2)	n/a	n/a
	PD-L1 IC2/3	42.0 (23.1-NE)	13.5 (8.8-26.3)	n/a	n/a
HRs for OS	BEP	0.59 (0.42–0.83)		1.38 (0.93–2.05)	
	PD-L1 IC0/1	0.75 (0.49–1.16)		n/a	
	PD-L1 IC2/3	0.51 (0.30-0.85)		n/a	

Where BEP is categorised as the intention-to-treat population with baseline plasma samples evaluated for ctDNA status.

*OS,* overall survival, *ctDNA* circulating tumour DNA, *RCT* randomised controlled trial, *MIUC* muscle-invasive urothelial carcinoma, *BEP* biomarker-evaluable population, *ITT* intention-to-treat, *CI* confidence interval, *IC* immune cell score, *NE* not estimable, *NR* not reached *PD-L1* programmed death-ligand 1.

### Impact of adjuvant immunotherapy use and further explorations

An overview of key trial data from all three trials CheckMate274, IMvigor010 and AMBASSADOR investigating the use of adjuvant immunotherapy in patients with high-risk MIUC is shown in Table 1, highlighting differences in trial endpoints as well as common findings. Overall, while CheckMate-274 and AMBASSADOR demonstrate increased DFS with ICI use over placebo, including the first OS benefit favouring nivolumab, IMvigor010 shows no DFS or OS benefit with atezolizumab use as an adjuvant ICI over placebo in the ITT population, which confounds the overall clinical efficacy of adjuvant ICI use.^19–23^ This is reflected in the current regulatory approvals of adjuvant ICI therapy for MIUC that are limited to adjuvant nivolumab and pembrolizumab.^24–28^ Notably, OS benefit with adjuvant ICI use is suggested in later updated analysis of IMvigor010, yet this is seen under exploratory analysis, which is more hypothesis-generating and requires caution against generalisation of results for adjuvant ICI's clinical efficacy.^22^ More so, OS data immaturity of trials limits the extent of OS analysis that can be performed and validity of overall findings, although data trends are nonetheless insightful and may still be commented on. In particular, for the AMBASSADOR trial, mature OS data remains yet to be published, in part due to lower-than-intended enrolment at 96% and censoring of patients that occurred due to regulatory approval of adjuvant nivolumab for MIUC during AMBASSADOR's trial rollout.^23^ More so, possible effects of off-trial ICI use in patients from the observation arm of AMBASSADOR are likely to contribute to further confounding of OS signal in the trial, further limiting trial endpoint interpretation of adjuvant pembrolizumab for MIUC.

Focus of the aforementioned trials on exploring ICI therapy in the adjuvant MIUC setting has led to ICI's increasing incorporation, notably with regulatory approval of adjuvant nivolumab for MIUC and NCCN guidelines’ incorporation of adjuvant pembrolizumab for MIBC, yet has also prompted further investigations into perioperative immunotherapy strategies for optimisation of MIUC care. In particular, the addition of ICI as both a neoadjuvant and adjuvant adjunct (a form of “sandwich” immunotherapy approach) to existing treatment strategies was examined in NIAGARA, a randomised phase 3 trial that was the first to investigate the use of anti-PD-L1 ICI durvalumab as a peri-operative ICI combined with NAC in cisplatin-eligible patients with muscle-invasive bladder cancer (MIBC), following the positive findings of DFS benefit seen with ICI monotherapy use in CheckMate-274 and AMBASSADOR.^37^ A total of 1063 patients with MIBC were randomised to receive neoadjuvant durvalumab and NAC for 4 cycles prior to radical cystectomy together with adjuvant durvalumab for a further 8 cycles, or NAC and radical cystectomy alone. The trial results show that the addition of perioperative durvalumab to the standard treatment regimen led to statistically significant improvements in the primary trial endpoint of event-free survival in MIBC patients treated with the addition of perioperative durvalumab compared to controls, demonstrating a 32% reduction in risk of disease progression, recurrence, not undergoing surgery, or death (HR 0.68; 95% CI, 0.56–0.82, p < 0.0001).^37^ More so, results from secondary endpoint of OS also suggest a 25% reduction in risk of death with durvalumab-based perioperative regimen compared to NAC and surgery alone (HR 0.75; 95% CI, 0.59–0.93, p = 0.0106), and overall, the trial findings support rationale for the use of a perioperative approach of immunotherapy in these cancers.^37^ Notably, NIAGARA findings led to recent regulatory approval by both the FDA and the European Commission of the “sandwich” perioperative durvalumab-based strategy, including neoadjuvant durvalumab combined with NAC, followed by single agent durvalumab as adjuvant treatment following radical cystectomy, for MIBC patients.^38,39^ Beyond exploring adjuvant immunotherapy use, seen in CheckMate274, IMvigor010 and AMBASSADOR, NIAGARA highlights “sandwich” perioperative strategies that may be of further interest to explore with immunotherapy use in MIUC, especially given that its investigation into the use of neoadjuvant ICI alongside adjuvant ICI, thought to help prime anti-tumour immunity and eradicate micrometastatic disease before and after surgery, has demonstrated feasibility and efficacy with tolerability and no new safety signals.^37^

Further such explorations of perioperative strategies for MIUC have also notably included the Phase 3 KEYNOTE-905/EV-303 trial, for which first interim analysis supports a perioperative approach of combining ICIs with the more novel treatment strategy of antibody-drug conjugates (ADCs) for improving survival in patients with MIBC not undergoing cisplatin-based chemotherapy.^40,41^ The use of ADCs, i.e., tumour-specific monoclonal antibodies linked to cytotoxic agents for targeted tumour killing, has in recent years shown significant successes as a treatment choice for later-stage UC, as exemplified by trial findings and later approvals of the use of ADC enfortumab vedotin (EV) as both a monotherapy and in combination with pembrolizumab for locally advanced or metastatic UCs.^42–46^ Based on these encouraging results of perioperative ICI and ADC use prolonging survival for UC in the metastatic setting, KEYNOTE-905/EV-303 was designed to evaluate perioperative pembrolizumab combined with EV compared with surgery alone in earlier-stage UC settings, focusing on patients with MIBC not receiving cisplatin-based chemotherapy.^40,41^ Adults with MIBC (≥50% urothelial histology, T2-T4aN0M0 or T1-T4aN1M0 disease) who were cisplatin-ineligible or declined cisplatin were randomised either to the control group, receiving surgery (radical cystectomy and pelvic lymph node dissection) alone, or to the treatment arms, which involved 3 neoadjuvant cycles of pembrolizumab with or without EV followed by surgery and continued with 14 adjuvant cycles of pembrolizumab +/- 6 cycles of EV.^40,41^ Key findings suggest that adding pembrolizumab and EV to surgery as a perioperative treatment significantly improved survival for patients with MIBC who did not receive cisplatin-based chemotherapy; both the primary endpoint, EFS (NR versus 15.7 months; HR 0.40; 95% CI 0.28–0.57; p < 0.0001), and OS (NR versus 41.7 months; HR 0.50; 95% CI 0.33–0.74; p = 0.0002) showed notable improvements with the perioperative combined strategy compared to surgery alone.^40^ Importantly, this trial forms one of the first to demonstrate both an EFS and OS benefit in cisplatin-ineligible MIBC patients with this treatment approach, including across key predefined subgroups, and, supported by its manageable safety profile, shows promise of perioperative EV + pembrolizumab potentially representing a new standard of care for the high-need population of cisplatin-ineligible patients with MIBC as further analyses of KEYNOTE-905/EV-303's are awaited. Thus, overall, explorations of “sandwich” perioperative immunotherapy approaches in MIUC merit consideration and further research, presenting opportunities to expand the clinical use of ICIs already approved for use in MIUC.

## UTUC versus bladder cancer: should tumour subtype guide adjuvant therapy?

Given UC's categorisation based on tumour origin, questions have arisen on whether treatment efficacy may be affected by the tumour subtype (upper tract or lower tract disease) of MIUC patients. This is particularly relevant because, while UTUC and BC form part of the same overarching disease, they are also distinct, not just in origin but also in disease course and character, which may affect treatment response.^47^ Pathologically, UTUC patients have a higher frequency of muscle invasiveness at diagnosis (around 2/3rd) compared to in BC, where a lower percentage of 15–25% of patients are diagnosed with muscle-invasive de novo BC, with population-based studies conducted across Europe confirming higher prevalence of muscle-invasive disease in UTUC versus BC.^2,7,8,48,49^ More so, UTUC characterisation studies reveal notable differences in its tumour characteristics in comparison to lower tract disease, including mutational burden in UTUCs than seen for bladder cancer.^50,51^ This can be relevant when considering clinical efficacy of ICI use in UTUC subgroups as studies show that ICI-derived treatment benefit is associated with higher tumour mutational burden (TMB), linked to greater tumour antigen production, which in turn allows for better immune recognition and response potentiated by ICIs.^51–53^ In addition, subtypes within UTUC have also become apparent via whole-exome sequencing, featuring distinct genetic and molecular profiles. For example, UTUC can be classified into low-grade tumour, enriched for activating FGFR3 mutations and depleted of P53/MDM2 mutations, versus high-grade tumours that frequently show mutations in TP53 signalling, which are associated with worse prognosis and survival.^50,54^ Therefore, UTUC has notable features that may be relevant in impacting adjuvant treatment response for MIUC patients. Within the metastatic UC setting, results from meta-analyses find no significant benefit in treatment outcomes with ICI use for UTUC subgroups over controls, further prompting investigations into whether differential efficacy of adjuvant ICI use may also be seen for MIUC patients.^55^

Subgroup analyses of UTUC and BC for all three key phase 3 trials indicate that patients with UTUC did not show an improvement in clinical outcomes over control groups, whereas subgroup data was suggestive of possible clinical benefit with ICI use for patients with BC compared to controls in CheckMate-274 and AMBASSADOR (Table 1).^19–23^ Recent meta-analyses summarising the data on subgroups by tumour location across the trials, show with concordance that no significant improvement in DFS was suggested by data for the upper tract subgroup, in contrast to patients with lower tract tumours where DFS was calculated to improve by around 29%.^56–58^ However, while overall suggestive of an adjuvant treatment benefit restricted to lower tract over upper tract disease, these findings across all the trials are merely speculative, lacking validity, statistical significance and adequate statistical powering, in part due to small subgroup numbers (see Table 1) and non-specification of subgroup analysis, and thus it is advised against drawing generalised conclusions on the differential efficacy of adjuvant ICI use by MIUC tumour subtype based on these trials’ subgroup data alone. As seen in current clinical practice, there is no restriction on the use of the widely-approved adjuvant ICI, nivolumab, for MIUC patients based on whether their urothelial cancer is of upper tract or lower tract origin.^24–26^ However, NCCN guidelines for the US note adjuvant pembrolizumab as an available therapy option specifically for MIBC, i.e., lower tract disease.^28^ This is despite AMBASSADOR demonstrating DFS benefit with adjuvant pembrolizumab for MIUC as a whole and subgroup findings on differential efficacy of ICI based on upper or lower tract disease being speculative only.^23^ Thus, currently, there is limited basis from the three aforementioned key trials for restricting adjuvant ICI use in MIUC based on tumour origin alone.

Regardless, it is pertinent to highlight a stark difference in the inclusion of tumour subtype populations across the aforementioned trials; in comparison to CheckMate-274 and AMBASSADOR, which include similar proportions of patients with UTUC in their trial designs, with 21% and 22%, respectively, the inclusion of UTUC was limited to 7% in IMvigor010, the only of the aforementioned trials to fail to meet its initial primary endpoint.^19,21,23^ Whether differing proportions of UC subtype populations may have had an impact on overall adjuvant ICI efficacy for the trials is difficult to determine but nonetheless important to explore, especially given the contrasting treatment outcomes seen in the trials with adjuvant ICI use for MIUC (Table 1). Ideally, adjuvant ICI use should be examined in trials with larger patient cohorts of UTUC and lower tract disease, adequately powered and with prespecification of their subgroup analysis compared to controls, in order to examine whether these speculations of differential efficacy hold valid.

## Implications on clinical application of biomarkers in urothelial carcinoma

Beyond the traditional application of biomarkers for prognostication of patient survival, an additional area of excitement has been the growing use of biomarkers for prediction of treatment efficacy, offering insight into what therapies might be most appropriate for specific patient cohorts when guiding treatment choice. The prognostic use of biomarkers has also expanded, with their increasing application for the monitoring of disease status and the detection of recurrence, areas that are of key importance for high-risk MIUC patients who traditionally see high rates of relapse.^2,5,11^ Therefore, investigation of the use of biomarkers is key in examining prognostic and/or predictive functions that may help to improve treatment outcomes for MIUC patients in future practice. Notably, the two key biomarkers explored thus far in the aforementioned key trials investigating the use of adjuvant immunotherapy for MIUC include tumour PD-L1 expression, a widespread cancer biomarker, and ctDNA, an emerging tumour biomarker that offers analysis of tumour status by blood rather than tumour biopsy alone.

### PD-L1 tumour status: to guide or not to guide

PD-L1 tumour status use across several cancers, including metastatic UC, has shown beneficial use as a biomarker, especially in the context of treatment with ICIs that can target PD-L1, yet its utility as a prognostic and/or predictive biomarker in the setting of MIUC has shown conflicting results across the key phase 3 trials exploring adjuvant immunotherapy use.^31,32^ While CheckMate-274 demonstrates clear benefits in both DFS (HR 0.52; 95% CI, 0.35–0.72) and OS (HR 0.56; 95% CI, 0.36–0.86) with the use of adjuvant nivolumab among patients with a PD-L1 expression of 1% or more over the control groups, PD-L1 positivity in AMBASSADOR and IMvigor010 did not confer a treatment benefit in DFS, although both show PD-L1 status to be prognostic of worse outcomes, including recurrence and/or death.^19–22^ However, further trial updates of IMvigor010 suggest that among the subgroup of ctDNA-positive patients, a PD-L1 status of IC2/3, defined as PD-L1-expressing tumour-infiltrating immune cells covering ≥5% of the tumour area, is associated with longer OS survival over the control group (HR 0.51, 95% CI 0.30–0.85]) (Table 2), further confounding overall perspectives of the effect of PD-L1 tumour status on treatment benefits.^23^ The uncertainty of the importance of PD-L1 status effect on clinical outcomes is also reflected in the discrepancies seen in the criteria of the different regulatory approvals for adjuvant immunotherapy nivolumab; while, under the FDA, nivolumab is offered as an adjunct to all high-risk MIUC patients following surgical resection based on its demonstrated clinical benefit irrespective of PD-L1 status, this is limited to MIUC patients with a tumour PD-L1 expression of 1% or more by the European Commission.^24,25^ Comparatively, under the NCCN's recommendation of adjuvant pembrolizumab for MIUC, patients without prior NAC are eligible regardless of PD-L1 status, as reflected in AMBASSADOR's finding of DFS benefit irrespective of PD-L1 status.^23,28^ Several meta-analyses evaluating the overall effect of PD-L1 status on treatment benefit show that clinical benefit was seen irrespective of tumour PD-L1 status, with PD-L1 + ve status not providing any additional benefit over controls, suggesting that PD-L1 application, while shown across the trials to have prognostic value, is limited in its validity as a predictive biomarker for ICIs.^31–33^

However, arguably the differences across the trial designs for the assessment of PD-L1 status as a biomarker preclude direct comparison of trial results and collective analysis of PD-L1's impact on treatment outcome. Instead, it can be argued that the utility of PD-L1 status as a biomarker can only validly be assessed in the context of the trial design it was investigated in, which provides some clinical rationale for the EC regulatory approval's criteria restriction based on PD-L1 subgroup.^25^ One of such key differences seen in trial design across the key studies was the use of different ICI classes: anti-PD1 inhibitors nivolumab and pembrolizumab were the choice of ICI therapy investigated in CheckMate-274 and AMBASSADOR, respectively, whereas atezolizumab, a type of anti-PD-L1 inhibitor, was under investigation in IMvigor010.^19–23^ Whether ICI class of the adjuvant immunotherapy used in the trials may have affected the treatment predictive nature of PD-L1 status is uncertain; current overall findings, however, did not reveal a pattern in the results of PD-L1 as a predictive biomarker based on ICI class and further testing of additional ICIs and/or their wider application to the neoadjuvant as well as adjuvant setting of MIUC are needed to see if any pattern may become apparent. Another key difference in trial design pertains to the broad variety in the definitions and methods of assessment of PD-L1 status used across the trials, which limits inter-trial comparison of PD-L1's application as a biomarker. CheckMate-274 stratified PD-L1 status by tumour proportion score (TPS), there defined as the percentage of tumour cell membranes staining PD-L1 positive in a minimum of 100 tumour cells by immunohistochemistry (IHC), into PD-L1 expressions of >=1% versus <1%.^19^ In comparison, PD-L1 status in AMBASSADOR was assessed via combined positive score (CPS), which covers PD-L1 expression on both the tumour cells and immune cells in tumour environment, and for which higher PD-L1 expression was denoted by a positive CPS score, indicating that a tumour has enough PD-L1 to benefit from immunotherapy.^21^ Further difference is seen in IMvigor's evaluation of PD-L1 status, for which higher PD-L1 expression was based on the amount of PD-L1-expressing tumour-infiltrating immune cells detected by IHC to cover >=5% of the tumour area, represented as immunohistochemistry IC2 or IC3 status.^23^ Therefore, as highlighted, the trials varied greatly in their assessment of PD-L1 status, including from which cells PD-L1 expression was measured and by which thresholds positive PD-L1 status was defined, which confounds overall implication of the use of PD-L1 biomarker in this setting.

Of additional consideration is that immunohistochemical assessment of biomarkers in resected tissue, as standardly used for PD-L1 assessment, provides only a snapshot of biomarker status in time, in comparison to biomarkers for which continuous monitoring can be facilitated. This does not allow for potential changes in PD-L1 status over the trial course to be taken into account in the assessment of PD-L1's treatment predictive or relapse-prognostic functions, limiting the biomarker's clinical application.

Overall, trial findings support PD-L1 status use for prognosis, independent of therapy, yet whether its use extends to prediction of treatment benefit remains unclear; more uniformity in methods of PD-L1 status assessment would allow for more valid comparison across studies and clearer outcomes on the utility of PD-L1 as a predictive biomarker, including whether it should be used to guide adjunct ICI use to relevant patient subgroups.

## The growing use of ctDNA biomarker and its paradigm shifts in clinical practice

Following the discovery of the presence of tumour-derived DNA within plasma cell-free DNA (cfDNA) by Stroun et al. in 1989, an era of circulating tumour DNA (ctDNA) application has emerged within oncology, ranging from its use for the investigation of molecular drivers of cancer and the development of targeted therapy to its application for the monitoring of tumour burden and progression via tumour liquid biopsies.^59,60^ ctDNA represents DNA derived from tumour cells, typically shorter in length (145 bp) than physiological cfDNA of 166 bp length, that can be released from cells via apoptosis, necrosis and/or secretion.^61^ Plasma represents the most common source from which patient tumour ctDNA is derived, and presents a novel alternative to the standard of tissue biopsies that have traditionally been used to characterise cancers. Additional forms of liquid tissue biopsy are also becoming increasingly available, including tumour-derived DNA sourced from urine rather than plasma- an alternative that is particularly relevant in the context of urological cancers.^32,62^ Landmark use of ctDNA was first seen in the 2000s in the field of lung cancer, particularly non-small cell lung cancer (NSCLC), which was the first malignancy for which measurement of ctDNA was clinically approved for use in mutational testing of key actionable driver mutations, i.e., EGFR, which could be used for guiding targeted therapy, like EGFR tyrosine kinase inhibitors gefitinib and erlotinib that were used in patients with advanced NSCLC.^34,63,64^ Colorectal cancer (CRC) is another notable field in which use of ctDNA biomarker has been widely adopted, providing not only reliable biomarker detection for metastatic colorectal cancer, but also commercial utilisation for treatment guidance on adjuvant therapy based on ctDNA-based minimal residual disease (MRD) detection.^65–67^ Thus, the expanding application of ctDNA across several cancers, particularly in helping to guide adjuvant treatment decisions, has also prompted interest into its utility for urothelial carcinoma. Within the metastatic setting, ctDNA is already showing various promising applications; like in NSCLC, use of ctDNA has been applied to help to map the landscape of driver mutations of primary tumours in mUC, and, more so, reduction in ctDNA levels have been shown to be associated with favourable clinical outcomes in mUC patients.^68–70^ There is growing interest in the potential of ctDNA application for prognostication and for helping to predict treatment response, particularly within the MIUC setting, which sees high-risk of disease recurrence and for which monitoring of disease status in patients and appropriate tailoring of available adjunct therapies would benefit treatment outcomes.

### Prognostication and disease monitoring

The exploratory analysis of the IMvigor010 trial notably investigated the impact of ctDNA status for MIUC patients, demonstrating a prognostic role of ctDNA, with ctDNA positivity at the beginning of adjuvant atezolizumab treatment shown to be associated with poorer prognosis (HR 6.3; 95% CI, 4.45–8.92; p < 0.0001).^23^ Other studies similarly show that increased levels of ctDNA correlate with disease burden and predicted disease recurrence in bladder cancer.^71–73^ For example, one study revealed ctDNA positivity prior to and after standard care treatment to be highly prognostic of recurrence, with 76% of ctDNA + ve patients experiencing recurrence over a median follow-up of 21 months compared to 11% seen in ctDNA-ve patients.^73^ ctDNA-guided prognostication has also demonstrated great value in helping to monitor for disease recurrence, providing comparable or even better relapse detection over radiographic detection. A study by Lindskjrog et al. was one of the first to report the performance of minimal residual disease (MRD) testing using tumour-informed ctDNA, measured via high-sensitivity assays, in a prospective cohort of MIUC patients.^74^ A total of 68 NAC-naive, MIUC patients underwent serial ctDNA sampling before and after radical cystectomy over more than 5-years of follow-up, in helping to investigate the use of ctDNA as a prognostic predictor of MIUC relapse. The accumulated ctDNA analysis reveals that ctDNA levels helped to identify metastatic relapse with a sensitivity of 94% and a specificity of 98%, with a positive lead-time of 118 days for ctDNA-based relapse detection over radiographic detection.^74^ These findings suggest that the use of ctDNA can provide improved monitoring of MRD and relapse over traditional radiographic imaging, which can be limited in their sensitivity and lag time in diagnosing recurrent lesions.^33^ For such MRD monitoring methods to be implemented, however, not only must MRD be detectable at high-enough sensitivity and specificity but also have effective treatments available for durable eradication of the disease. With recent expansion of the adjuvant treatment setting of MIUC through inclusion of adjuvant immunotherapy strategies, this increases the feasibility of the incorporation of ctDNA biomarker testing for such application. Overall, current studies suggest a promising application of non-invasive ctDNA plasma assays for detection of disease recurrence alongside or possibly even in place of traditional imaging for future clinical practice. However, further recurrence monitoring studies are required to provide a reliable picture of overall detection sensitivity and specificity in ctDNA's comparison to the current standard of radiological cancer imaging, in order to implement ctDNA biomarker into future clinical practice for MIUC settings.

### Prediction of treatment benefit

Alongside its potential use for prognostication and disease monitoring, ctDNA biomarker application has also shown promise in the prediction of treatment effect, which is key for helping to guide specific therapies to appropriate patient groups. Results from the exploratory analysis of IMvigor010 have been the first to demonstrate clinical benefit derived with adjuvant atezolizumab use in ctDNA + ve patients, particularly in OS (HR 0.59, 95% CI, 0.41–0.83) (Table 2).^23^ Comparatively, increases in DFS and OS with atezolizumab were not seen for ctDNA-ve patient cohorts. Of important note, the clearance of ctDNA achieved with adjuvant ICI was shown to be associated with better clinical outcomes of longer OS, with OS of 19.9 months at <50% clearance (95% CI, 16.4–32.2) compared to an OS of 60 months at 100% ctDNA clearance (95% CI, 35.5-not estimable).^23^ These improvements in survival outcomes for high-risk MIUC patients that achieved ctDNA reduction from atezolizumab use corroborate results seen in other settings of atezolizumab use for ctDNA + ve patients; the phase 2 ABACUS trial investigating neoadjuvant atezolizumab use in MIUC patients, similarly finds that patients with ctDNA clearance or reduction with atezolizumab showed improvements in survival outcomes versus observation.^75^ However, while the IMvigor010 trial results suggest that ctDNA positivity in MIUC could help to predict treatment benefit with use of adjuvant atezolizumab, important for tailoring therapy, the retrospective exploratory nature of the IMvigor010 study means that the results are only hypothesis-generating and further studies are needed to explore these possible findings. Tailoring of adjuvant treatment for MIUC patients based on liquid biopsy ctDNA status would offer a non-invasive, accessible method of optimising individual treatment outcomes while limiting additional treatment and experience of associated side-effects to those patient populations most likely to benefit, rather than in patients without positive predictive biomarker status.

### Exploration of adjuvant treatment guidance for MIUC

Of further studies currently ongoing to explore ctDNA's biomarker role in MIUC, the IMvigor011 trial follows on from exploratory analysis of IMvigor010, and is one key phase 3 trial aiming to evaluate both ctDNA-guided treatment with adjuvant atezolizumab compared with placebo in patients with high-risk MIBC and ctDNA-based surveillance.^70^ In this international, randomised, phase 3 trial, patients with high-risk MIBC (>=ypT2 UC and/or N + after NAC, and pT2 UC and/or N + without prior NAC) are followed by serial plasma ctDNA analysis (tumour-informed Signatera assays) every six weeks as they enter a surveillance phase or until detection of ctDNA positivity, at which point patients are randomised (2:1) to treatment with atezolizumab or placebo for up to 12 cycles/1 year or until recurrence.^76^ This ongoing study aims to examine the impact of ctDNA-guided treatment on DFS for MIBC patients with detection of ctDNA positivity up to 20 weeks after cystectomy as a primary trial endpoint, offering more direct evaluation of ctDNA's utility in guiding adjuvant ICI treatment. More so, exploratory analysis of the trial's surveillance cohort reveals preliminary findings on the use ctDNA-assisted disease monitoring; early data suggests that patients who remain persistently ctDNA-negative after surgery, i.e., MRD-negative (as confirmed by radiologic imaging), have a very low risk of recurrence and could potentially avoid adjuvant treatment, reporting DFS and OS rates of 88% and 98% at 18 months, respectively, for the ctDNA-negative 171 patients included in the analysis.^77^ Notably, the latest findings of the primary analysis of IMvigor011 presented in October 2025 demonstrate that the ctDNA-guided treatment approach is associated with significant improvements in survival outcomes for patients with MIBC.^78^ In ctDNA-positive patients it was found that adjuvant atezolizumab reduced the risk of disease recurrence or death by 36% compared to placebo at a median follow-up of 16.1 months (HR 0.64; 95% CI, 0.47 to 0.87; p = 0.0047). Similarly, OS results show statistically significant improvements with adjuvant atezolizumab use in ctDNA-positive patients compared with placebo, demonstrating a median OS of 32.8 months for patients on atezolizumab versus 21.1 months for placebo (HR for death, 0.59; 95% CI, 0.39 to 0.90; p = 0.0131).^78^ Not only does the latest update suggest survival benefits for ctDNA-positive patients treated with adjuvant atezolizumab, but it also continues to show excellent outcomes for monitored ctDNA-negative patients without additional immunotherapy; in the 357 patients that persistently tested ctDNA-negative, the DFS rate was 95.4% at 1 year and 88.4% at 2 years, with similarly good outcomes for OS rates, showing 100% and 97.1% at 1 and 2 years, respectively.^78^ Therefore, while further trial updates are awaited, initial results of IMvigor011 are promising and suggest that serial ctDNA testing may have greater clinical utility than landmark ctDNA testing as a risk stratification tool and lend increasing confidence to the use of ctDNA status for guiding the need for or sparing of additional treatment in the adjuvant setting.^76,77^

The TOMBOLA study is another ongoing, national, non-randomised phase 3 trial evaluating whether serial ctDNA testing could be used to identify bladder cancer patients that might benefit from adjuvant atezolizumab.^79^ Patients with MIBC (cT2-4a UC) following NAC and radical cystectomy undergo monthly plasma ctDNA analysis (tumour-informed ddPCR) and are commenced on 1-year treatment with atezolizumab upon detection of ctDNA positivity, with complete response after treatment assessed as the primary endpoint, alongside OS, duration of freedom from clinical relapse and cancer-specific survival as secondary trial endpoints. Based on preliminary results from the trial thus far, serial ctDNA testing is shown to help to identify patients who might benefit from early immunotherapy at a time of minimal metastatic disease, picking up 101 (56%, 101/179) ctDNA-positive patients after radical cystectomy, with 65% detected within 4 months after surgery.^80^ Of the consistently ctDNA-negative patients, only 2 (3%) developed metastases on CT scans during follow-up, and overall shows promise of ctDNA testing for treatment de-escalation (in ctDNA-negative patients) and patient selection for adjuvant therapy (in ctDNA-positive patients). Similarly, another notable trial studying treatment intensification and de-escalation based on ctDNA and corresponding MRD status is the randomised, phase ⅔ MODERN trial, currently still recruiting, which also aims to evaluate ctDNA-guided adjuvant treatment, notably regarding the use of already-approved adjuvant nivolumab, in patients with MIBC.^81^

Overall, the use of ctDNA in MIUC provides an avenue for paradigm shifts in the monitoring and therapy guidance of MIUC's adjuvant therapy landscape, with its pioneering use as a biomarker as already seen for NSCLC and colorectal cancer. ctDNA analysis, with its many aforementioned applications across the neoadjuvant and/or adjuvant setting, is particularly advantageous in its minimally invasive nature of sampling, i.e., blood samples for plasma ctDNA analysis, which offers wide availability to patients and the opportunity for serial sampling. However, disadvantages in its application also need to be recognised to focus on how these areas may be optimised. One key limit to the detection of plasma ctDNA is the amount of ctDNA released into the bloodstream, a limiting factor to analysis that can vary from patient to patient, influenced by other factors like tumour stage and volume (where earlier stages and lower volumes show lower ctDNA) as well as biological patient features like age, obesity and comorbidities.^33,82^ Focus is shifting towards maximising other preanalytical factors like volume of plasma collected, use of appropriate collection kit and storage to preserve the integrity of ctDNA as well as rapid processing of the samples.^33,83^ More so, research is also ongoing into more powerful ctDNA detection methods to help detect even low-abundance (<10 ng) ctDNA for better sensitivity in patients, especially relevant for those whose personal and/or tumour characteristics are associated with suboptimal tumour ctDNA shedding.^33^ Recent developments include the wider application of high-sensitivity detection assays, such as used by Christensen et al., for ultra-deep sequencing of plasma ctDNA in patients with urothelial bladder carcinoma to facilitate MRD monitoring.^73,76,77,79^ Notably, these types of ctDNA detection assays, including the assays used in the aforementioned ctDNA trials, are based on tumour-informed ctDNA analysis, i.e., personalised ctDNA assays identified by sequencing of a patient's own tumour rather than by a pre-defined panel of common cancer-associated mutations, which studies, including in other cancer settings, like CRC, suggest are associated with increased sensitivity over tumour-agnostic ctDNA analysis^73,84^ Furthermore, other outstanding questions regarding ctDNA analysis relate to the optimal timing, frequency and positivity threshold to be used for ctDNA analysis.^33,74,83^ Results from further studies are needed to determine the optimal threshold at which ctDNA positivity is best categorised, intervals at which ctDNA sampling should be carried out as well as the different threshold levels that correspond to clinically meaningful correlates. In particular, the timepoints of longitudinal monitoring are of key consideration that can affect sensitivity; this is particularly highlighted by IMvigor011's preliminary findings that suggest that serial ctDNA testing may have greater clinical utility than landmark ctDNA testing in detecting MRD and a risk stratifying.^79^ Additionally, the role of urinary ctDNA alongside or in comparison to plasma ctDNA for urological cancers requires further exploration; while plasma ctDNA presents the current liquid choice of analysis due to optimal isolation and collection protocol and its current variety in application, some studies suggest that urine ctDNA better reflects overall tumour burden and clonal evolution of early-stage bladder cancer compared to plasma ctDNA, and may provide a better avenue for disease monitoring in earlier, non-muscle invasive disease and bladder-preserving approaches.^32,33^ Further studies exploring their distinct roles are needed to see whether ctDNA analysis itself may also be tailored by liquid source for UC patients according to disease stage and the intended application.

## Conclusion

To conclude, adjuvant immunotherapy for high-risk, muscle-invasive urothelial cancers presents a promising area, supported by regulatory approval for both nivolumab and pembrolizumab, and further trials exploring their tailored use among MIUC patients. While only PD-1 inhibitors nivolumab and pembrolizumab have been shown to improve clinical outcomes in DFS and (projected) OS in the treatment groups, benefits are shown with anti-PD-L1 therapy atezolizumab in patients with greater ctDNA, highlighting ctDNA's utility as predictive biomarker alongside its use in prognostication. Comparatively, the value of the use of PD-L1 status in MIUC remains contentious with conflicting results from the trials on their prognostic or predictive value, thus favouring the use of ctDNA as a pioneering biomarker to be further explored in this setting. The impact of tumour subtype (UTUC or lower tract disease) on the efficacy of adjuvant ICI treatment remains unclear, given the lack of statistical power in analyses thus far. Data from further larger trials is needed to inform whether adjuvant therapy may best be tailored to particular patient subtypes, with the ultimate aim to help provide more insight into optimisation of the use of adjuvant immunotherapy for patients with MIUC care in order to improve treatment outcomes.
